# The impact of post-encoding alcohol consumption on episodic memory recall and remember-know responses in heavy drinkers

**DOI:** 10.3389/fpsyg.2023.1007477

**Published:** 2023-03-07

**Authors:** Benjamin Butterworth, Christopher James Hand, Karen Lorimer, Julie Gawrylowicz

**Affiliations:** ^1^Glasgow Caledonian University, Glasgow, United Kingdom; ^2^University of Glasgow, Glasgow, United Kingdom; ^3^Abertay University, Dundee, United Kingdom

**Keywords:** episodic memory, alcohol intoxication, psychological trauma, memory, alcohol, cognition, traumatic event

## Abstract

**Introduction:**

People often consume alcohol following trauma, particularly in response to distressing memories. To date, little is known about how post-encoding alcohol consumption influences episodic memory recall for negative events. Understanding these effects may help to improve support for trauma victims – for example, witnesses and victims of crimes.

**Methods:**

We tested 60 participants who self-described as heavy drinkers. After watching an analog trauma film, half were allocated to consuming a moderate dose of alcohol (Alcohol-Exposed group), while half received a placebo drink (Placebo-Control group). Immediately and after a one-week delay, participants recalled the event *via* free and cued recall tasks. Participants also gave remember-know responses and confidence ratings, elucidating alcohol’s effect on experiential memory.

**Results:**

Free recall performance was similar for the Alcohol-Exposed group and the Placebo-Control group during Sessions 1 and 2. The Alcohol-Exposed group benefitted more from the delayed repeated retrieval attempt. For the cued recall task, the Alcohol-Exposed group provided more “Do not Know” responses compared to the Placebo-Control group in both sessions. For the Alcohol-Exposed group only “Correct Know” responses increased from Session 1 to 2. Although memory performance improved across sessions, confidence levels decreased from Session 1 to 2 in the Alcohol-Exposed group.

**Discussion:**

Post-encoding alcohol consumption appears to impact immediate episodic memory retrieval; however, this effect is only temporary in nature. No evidence was found that alcohol primarily reduces remembering responses. Much like previous findings focusing on pre-encoding alcohol consumption (Hagsand et al., 2017), current findings suggest that providing individuals who drank alcohol after witnessing an incident with a delayed repeated retrieval attempt can lead to more complete and accurate testimonies.

## 1. Introduction

There is a strong link between exposure to trauma, such as violent offenses and serious injuries, and alcohol use. In Scotland, almost half (49%) of common assault records refer to the consumption of alcohol (National Statistics Publication for Scotland, 2015/16). Fekadu et al. (2019) found that 15% of road traffic accident survivors reported subsequent hazardous alcohol consumption. Similarly, Boscarino et al. (2006) found that citizens with greater exposure to the September 11 attacks in New York demonstrated higher alcohol consumption 1 and 2 years after the event happened. Similar links between trauma exposure and subsequently increased alcohol consumption were observed in survivors of child sexual abuse (Ullman, 2016), prisoners of war (Engdahl et al., 1998), and victims of natural disasters (Flory et al., 2009). Victims and witnesses may turn to alcohol after trauma to alleviate intrusive thoughts and memory flashbacks (Kaysen et al., 2006). It is therefore crucial to study how post-encoding alcohol consumption impacts episodic memory recall. Findings can inform treatment for individuals who experienced trauma and also provide more nuanced evidence-based guidance on how to best interview witnesses and victims. To the best of our knowledge, no experimental study has investigated the effects of alcohol on episodic memory *after* the experience of trauma. The current study fills this gap in the literature by examining the effect of post-encoding alcohol consumption on episodic memory recall and experiential recollection of a simulated severe car accident.

### 1.1. The impact of alcohol on memory for negative events

Alcohol can have diverse effects on memory performance (Gawrylowicz and Bartlett, 2021). If alcohol is consumed *before* the negative event took place, it often has a detrimental impact on one’s memory recall, especially on recall completeness (see Jores et al., 2019, for a review). For example, Bartlett et al. (2021) found that intoxicated mock witnesses provided significantly fewer details when recalling an opportunistic theft, but their accounts were as accurate as those of sober participants. Crossland et al. (2020) compared different interview types and tested mildly, moderately, and severely intoxicated individuals. Sober participants’ accounts of a mock theft were significantly more complete than those of severely intoxicated individuals.

It could be argued that the to-be-remembered events employed in some laboratory studies do not elicit the same negative emotions witnesses might experience when observing a real crime. For example, both Schreiber Compo et al. (2012) and Crossland et al. (2016) showed a non-violent simulated theft of IT equipment in a classroom setting. However, studies that have used more traumatic stimuli have revealed similar results. For example, Flowe et al. (2016) examined the influence of alcohol on remembering an interactive hypothetical sexual assault scenario. Intoxicated participants reported less information, but the provided information was not less accurate. Similarly, Hildebrand Karlén (2016) found that when questioned immediately, highly intoxicated mock-witnesses provided shorter accounts, but not less accurate accounts of an intimate partner violence scenario compared to moderately intoxicated and sober witnesses.

To study the memory-related effects of different types of aggressive contexts in relation to alcohol consumption, Hildebrand Karlén et al. (2019) examined intoxicated and sober participants’ accounts for neutral, verbally, and physically aggressive intimate partner violence contexts. Overall, alcohol decreased the number of gist details reported for all emotional contexts. However, a significant interaction between the degree of intoxication and emotional context was revealed. Sober and moderately intoxicated participants recalled most details from the verbally aggressive context, followed by the neutral context, and least gist details from the physically aggressive one; whereas severely intoxicated participants recalled most gist details from the neutral context, followed by the verbally aggressive one, and least from the physically aggressive one. Their findings suggest that alcohol-related memory deficits might be influenced by the emotional context of the to-be-remembered event.

Thus, a mild to moderate dose of alcohol consumed *before* the to-be-remembered event is encoded typically leads to less complete accounts, which are not necessarily less accurate. This seems to be true for both relatively neutral (e.g., opportunistic theft) as well as more traumatic events (e.g., sexual assault). The specific emotional content of the to-be-remembered event might further impact how much information will be remembered. Research examining the impact of pre-encoding alcohol consumption on mock-witness memory has expanded over the last decade, but research on the effect of post-encoding alcohol intoxication on eyewitness memory recall is lacking (see Hildebrand Karlén (2018) and Jores et al. (2019) for a tabular overview of studies on intoxicated witnesses’ recall alongside the specific to-be-remembered events employed).

### 1.2. Coping and post-traumatic alcohol consumption

A potential motivator for post-traumatic alcohol consumption is to cope with negative life experiences. Beseler et al. (2011) surveyed individuals about their drinking motives before the September 11 attacks and about subsequent alcohol consumption after the event. Drinking to cope with negative affect and drinking for enjoyment were the two motivators that significantly predicted increased alcohol consumption after the terrorist attacks. According to the self-medication hypothesis, individuals use substances, including alcohol, to help manage psychiatric symptoms, contributing to addiction over time (Khantzian, 1997). While originally focusing on clinical populations, the self-medication hypothesis has since been applied to trauma-exposed and heavy-drinking populations without clinical diagnoses (Luciano et al., 2020). In addition to negative affect, individuals use alcohol to “cope” with negative memories resulting from trauma. For example, daily intrusions and re-experiencing symptoms were specifically related to self-reported urges to drink and alcohol consumption among a sample of female college students with sexual assault experience (Kaysen et al., 2014). Stewart et al. (2004) investigated the relationship between drinking motivations and trauma symptoms relating to an aircraft disaster among a sample of emergency responders. Scores from the COPE scale (Carver et al., 1989), an instrument assessing coping styles, revealed that individuals experiencing the most frequent and severe PTSD symptoms following the disaster were those most likely to drink (or use drugs) with the specific intention to forget traumatic memories. While the study is somewhat limited by the small number of participants (*N* = 7), Miller et al. (2014) found a similar relationship between PTSD symptoms and alcohol use to forget in a larger sample of individuals suffering from social anxiety (*N* = 83). Only two items from the updated version of the drinking motivations questionnaire significantly predicted a diagnosis of alcohol dependency, including drinking to forget painful memories. Thus, post-traumatic alcohol use is common, and a main motivator is to cope with negative affect including forgetting traumatic memories. But does drinking to forget really work?

### 1.3. Post-encoding intoxication and memory

Up till now, few studies manipulated post-encoding intoxication (e.g., Gawrylowicz et al., 2017; Schreiber Compo et al., 2017; Doss et al., 2018). Bruce and Pihl (1997) asked 42 social drinkers to rate depressing, elating, and neutral statements while sober. Half of the participants then received alcohol and the other half a placebo. Participants’ memory for the statements was then tested 24 h later when all participants were sober again *via* an incidental free recall test. Participants who consumed alcohol after encoding but prior to recall had superior recall memory across statement types. Gawrylowicz et al. (2017) presented participants with a video of a mock theft *before* consuming alcohol or a placebo. Participants were then presented with misinformation in the form of a written narrative and their memory for the video was subsequently tested. Participants who consumed alcohol after watching the video but before encoding the misinformation were less likely to report misinformation in the memory test compared to sober participants. Gawrylowicz et al. argue that alcohol consumption might have decreased retrograde interference (Mueller et al., 1983). That is, intoxication reduces the formation of new memories thereby protecting already existing memories from the negative effects of misleading post-event information.

Schreiber Compo et al. (2017) tested state-dependent recall by providing participants with or without alcohol before watching a video about a mock theft, and again when retrieving the content of the video 1 week later. While intoxicated participants provided more complete testimonies when interviewed immediately, no effect for state dependency was observed. While the study did not specifically seek to test post-traumatic alcohol intoxication, it was found that participants who were sober at encoding and intoxicated 1 week later at retrieval reported less information than those who were sober at encoding or received a placebo at retrieval. The findings suggest that post-traumatic alcohol consumption (1 week later) does not affect free episodic memory recall.

Finally, Doss et al. (2018) reanalyzed data from studies testing how alcohol and similar sedatives affect recollection and familiarity when consumed at encoding or consolidation. Their findings suggest that alcohol and similar sedatives attenuate episodic memory performance when consumed prior to encoding but improve performance when consumed during memory consolidation. Post-encoding alcohol consumption increased both recollection and familiarity of neutral information but had no effect on recollection or familiarity estimates for negative or positive information. Like Hildebrand Karlén et al. (2019)’s findings, these findings suggest that in addition to the timing of alcohol consumption, the emotional content of the to-be-remembered information impacts subsequent episodic memory recall.

### 1.4. Remembering and knowing

While many researchers study alcohol-related effects on episodic memory accuracy and completeness, fewer investigated how alcohol impacts the experiential aspect of memory. According to Tulving (1985), the recognition of an earlier encountered item can be accompanied by two mental processes “remembering” and “knowing.” Whereas remembering is accompanied by conscious recollection and vivid re-experiencing of details associated with the studied item/event, knowing is accompanied by a feeling of familiarity and the absence of conscious recollection of contextual details. Gardiner and Richardson-Klavehn (2000) suggest that remembering and knowing are functionally independent of each other, as evidenced by research showing that different independent variables, such as drugs, can selectively impact one or the other (i.e., systematic dissociation). For example, Curran et al. (1993) found that lorazepam selectively impaired “remember” responses without impacting “know” responses. In a subsequent study, Curran and Hildebrandt (1999) tested the dissociative effects of alcohol on remember/know responses. Intoxicated and sober participants studied opposite word-pairs presented either in full (e.g., DAY – NIGHT) (“read” condition) or the first word was presented in full but only the first letter of the second word was presented (e.g., DAY – N_) (“generate” condition). Participants then took part in an old/new recognition test, including old and lure words, all presented in full. As with lorazepam, alcohol-reduced “remember” responses but not “know” responses, particularly in the “generate” condition.

So, does alcohol reduce detailed contextual recollection? That would be in line with the consistent finding from the eyewitness memory literature that alcohol negatively affects the completeness of memory accounts but not their accuracy (Flowe et al., 2021). In addition to studying how post-encoding alcohol intoxication affects memory completeness and accuracy for a negative event, we also examine how it impacts experiential recollection by collecting remember/know responses to get a better understanding of *how* alcohol impacts episodic memory.

### 1.5. Aims and hypotheses

The present study aimed to test the effects of post-encoding alcohol intoxication upon episodic memory recall for a negative event in a sample of heavy-drinking individuals. We use the term alcohol intoxication in this paper to refer to an individual’s temporal condition resulting from the acute administration of a low to moderate dose of an alcoholic beverage. The experimental group (Alcohol-Exposed group) received an alcoholic drink after watching an analog trauma film, while the control group received a placebo (Placebo-Control group) after viewing the same material. Participants, but not the experimenter, were blind, so did not know to which group (experimental vs. control) they belonged. Episodic memory accuracy and completeness were compared between groups both immediately and after a week’s delay using free and cued recall tests. In addition, experiential recollection was examined *via* “remember/know” responses. In line with earlier work on pre-encoding intoxication on eyewitness memory, we hypothesized that post-encoding alcohol consumption would have a negative effect on individuals’ immediate and delayed memory recall. This alcohol-related detrimental effect would be most pronounced for recall completeness. We also expected that alcohol would impact individuals’ remembering experience, leading to fewer “remember” responses and subsequently more “know” responses. If those alcohol-related effects are short-lived, then we would expect no differences between the Alcohol-Exposed group and the Placebo-Control group during the one-week delayed session.

## 2. Method

### 2.1. Design

A mixed-factors design was used, with an independent factor of alcohol intoxication condition (Alcohol-Exposed vs. Placebo-Control) and a repeated factor of testing session (Session 1 & Session 2). Participants were assigned using a random number generator to one of the two conditions. The dependent variables were the number and accuracy of details during free recall and “remember/know” responses and confident judgments during the cued recall.

### 2.2. Participants

Most research features social drinkers from student populations, whereas motivations for post-traumatic alcohol use differ among heavier drinkers (Dixon et al., 2009; Smith et al., 2018). We sampled from a population of heavier drinkers, who are more likely to be affected by post-traumatic alcohol use (Kaysen et al., 2006; Olff et al., 2007). Participants were recruited from the wider community and the Host University’s student population *via* adverts in bars and businesses, as well as local community groups on social media. Participants received a monetary reward and/or course credit for taking part. The sample contained a mixture of students and people from the general population, with a higher proportion of non-students (38%) compared to previous studies of pre-traumatic intoxication [e.g., 0% in Bisby et al. (2009); 20% in Jaffe (2018)].

An *a priori* power analysis was conducted with G*Power 3 (Faul et al., 2007) based on an anticipated independent t-test analysis. Given the large effect sizes observed in previous studies (Bisby et al., 2009, 2010), a sample size of 60 participants was targeted (generating an expected power = 0.92, given *α* = 0.05 and Cohen’s *d* = 0.8). Through advertising on campus and through the local community, 63 participants (29 females, 34 males) were recruited for the study; of those, 60 returned for the second session. Participants were all aged over 18 (*M*_age_ = 28.9 years, SD_age_ = 7.9) and self-described as individuals who drank heavily, which was defined as consuming 14 or more units of alcohol a week (as per NHS guidelines, 2018). Weekly alcohol consumption was assessed *via* self-report measures during screening, with the lead researcher providing participants with an infographic to help calculate alcohol units. All participants confirmed that they did not meet any of the exclusion criteria, including being diagnosed with an alcohol use disorder (AUD) or PTSD [assessed *via* the alcohol use disorder identification test (AUDIT) and the civilian version of the PTSD checklist (PCL-C)]; being pregnant; taking a medication that interacts negatively with alcohol; having been physically injured or psychologically affected by a car accident (as the experimental video stimulus depicted a traumatic car accident); being intoxicated at the beginning of the experiment (confirmed with a breathalyzer test).

### 2.3. Materials

#### 2.3.1. Screening tools

The fast AUDIT and PCL-C were administered to confirm that participants did not meet the criteria for AUD or PTSD. The fast AUDIT includes four self-report Likert scale questions about binge drinking, alcohol blackouts, impaired functioning due to intoxication, and concerns over drinking. The scores from these questions are combined to calculate a risk factor for AUD (i.e., low risk, high risk, and possible AUD). The fast AUDIT has been shown to be sensitive at identifying undiagnosed AUD across different settings (Hodgson et al., 2002). A score of 20 or greater is used as a cut off to exclude potential participants from harm.

The PCL-C is a 17-item Likert scale questionnaire indicating the severity of different clusters of trauma symptoms in the past month (e.g., experiencing intrusive memories, being easily startled, feeling distant from other people). The PLC-C has been found to be particularly effective and reliable when used in non-clinical samples (Conybeare et al., 2012). Scoring moderate symptom severity or higher across at least one item from each symptom cluster indicates the potential for a PTSD diagnosis. Prospective participants meeting these criteria were therefore excluded from the study, as their participant could exacerbate pre-existing symptoms of psychological trauma.

#### 2.3.2. Trauma film

Participants watched an analog trauma film from Strange and Takarangi (2012), adapted from the public service film “Cow” (2009). The 4 min and 16 s film dramatizes a car accident, showing gruesome imagery of emergency services treating injured people screaming in pain. The film has been used in previous research (e.g., Strange and Takarangi, 2012; Meyer et al., 2014) and has not been reported to cause lasting psychological distress, with intrusive memories typically subsiding after 1 week. The film includes a soundtrack with melodramatic music and uses a variety of different shots (e.g., first-person, third-person, birds-eye view, slow motion). While these features would not be present in real-life footage of a car accident, they do not prevent the film from serving its intended purpose (i.e., inducing intrusive memories and assessing episodic memory performance).

#### 2.3.3. Drinks and breath alcohol measurement

Participants in the alcohol condition consumed vodka diluted with tonic water and orange cordial (1:3 ratio). Alcoholic beverages were mixed using a blood alcohol level (BAL) calculator (Curtin, 2000) taking participants’ sex and body weight into account. The target BAL was 0.04% with a maximum dose of 150 ml of 37.5% abv vodka. Participants in the placebo condition received drinks containing tonic water and orange cordial equivalent in volume to the total volume of the alcoholic beverage to drink. All participants were asked to consume their beverages with a straw and vodka was smeared on the rim of each glass to disguise the smell of the drink. A handheld Lion alcometer 600 was used to measure participant’s Blood Alcohol Concentration (BAC) throughout the study.

#### 2.3.4. Distractor task

Participants engaged in a one-hundred-piece Disney Princess jigsaw puzzle to distract them from rehearsing the content of the trauma film and to allow the alcohol to absorb into the bloodstream. This lasted 45 min (i.e., 30 min drinking time plus 15 min for absorption of alcohol into bloodstream), before the experimenter asked participants to stop.

#### 2.3.5. Free recall task

Episodic memory performance was assessed by asking participants to write down as much as they could remember about the film within 5 min (consistent with previous studies, e.g., Bisby et al., 2010). Each participant was provided with four sides of lined A4 paper and a pen to write with (more paper was provided upon request).

#### 2.3.6. Cued recall task

Participants were presented with 16 questions about the film, with the researcher writing down each answer before progressing to the next question. Each question related to a perceptual detail from the film, featuring text (e.g., “*What did the road sign say*?”); colors (e.g., “*What was the color of the blanket used to cover the woman*?”); frequencies (e.g., “*How many fire engines can be seen at the collision site*?”); and temporal details (e.g., “*What type of emergency vehicle is the first to arrive at the scene of the collision?*”). A pilot study featuring eight participants was conducted, whereby participants were presented with cued recall questions after watching the trauma film. The pilot established that participants understood each question, that the questions were not too easy or difficult (i.e., avoiding ceiling and floor effects), and that answers to each question were sufficiently discrete (i.e., not subjective).

After answering each cued question, the remember-know procedure was employed (Yonelinas et al., 1998), whereby participants had to state whether they “remembered” their answer or if they felt like they “knew” the answer. The following definitions were provided to participants: “remember” refers to consciously recalling seeing that part of the film, whereas “know” refers to not remembering seeing that part of the film, but the detail is familiar. Participants were also instructed to provide a confidence rating for each answer, with 100 being completely certain and 0 being not certain at all. If participants could not provide an answer, their response was recorded as “do not know” with a confidence rating of zero.

#### 2.3.7. Online alcohol consumption diary

An online diary was created with https://esurv.org to record participants’ alcohol consumption between study sessions. Participants recorded different details about their daily drinking behavior: how much they drank, what they drank (e.g., beer, wine, and spirits), when they drank (e.g., afternoon, evening, and night), how they felt while they were drinking (e.g., happy, sad, and angry), and if there was a reason for drinking. Responses were recorded using a multiple-choice questionnaire (e.g., for alcohol consumption – I drank 0 drinks, 1–2 drinks, 3–4 drinks, and 4+ drinks). The highest option for number of drinks was capped at 4+, to minimize feelings of shame or guilt in participants. Most participants (92.5%) provided self-reported measures each day.

### 2.4. Procedure

#### 2.4.1. Session 1

Prior to commencing the first experimental session in the lab, participants were asked to confirm their eligibility by completing the screening questionnaires *via* email. Eligible participants were then scheduled for a face-to-face experimental session in the laboratory at the Host University campus. Participants took part individually. At the beginning of the session, participants read through the information sheet and provided consent. Participants then completed a breathalyzer test to ensure they were sober.

Participants were then presented with the trauma film. They were told to pay close attention to the film and that they could withdraw from the study and stop watching the film at any time. Thereafter, the beverage administration phase followed. Participants were provided with four beverages, which were prepared prior to participants’ arrival. The four beverages were either all alcoholic or all placebos. Participants were informed that they had to consume the drinks within 30 min but not faster than 20 min (see Gawrylowicz et al., 2017, 2019). Participants completed the distractor task while consuming their beverages and were then given an additional 15 min to complete the distractor to allow the alcohol to be absorbed. Next, participants engaged in two breathalyzer tests taken directly after each other to confirm whether they had reached their target BrAC (i.e., 0.20 mg/L alcohol: breath in the experimental condition, equivalent to a BAC of 0.04%). Participants were instructed to rinse their mouths with water before taking the first breathalyzer test to ensure that no residual alcohol would influence the accuracy of the reading. The mean average of the two measurements was used in the further analysis of data.

After taking their second breathalyzer test, participants completed the free recall and cued recall tasks. Upon completion, participants were breathalyzed again before leaving the laboratory. Participants were advised to stay behind until their BrAC has fallen to a safe level (i.e., 0.22 mg/L alcohol: breath) and were provided with snacks, drinks, and magazines in a comfortable area while passing the time. Participants who wished to leave before their BrAC has fallen to 0.22 mg/L alcohol: breath had to sign a disclaimer form, in addition to providing a final breathalyzer measurement. While a full debrief was not provided until the end of the second study session, any/all questions by participants were answered before concluding the first session.

#### 2.4.2. Session 2

**Figure 1:**
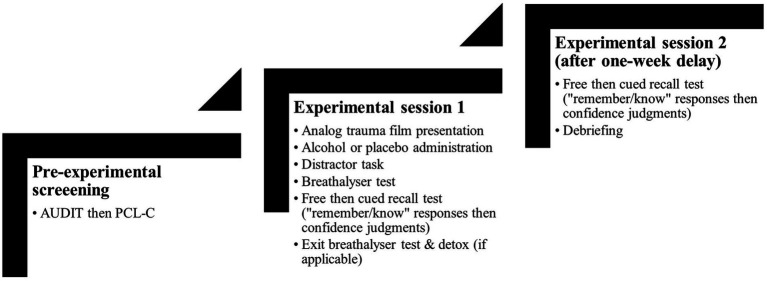
Schematic flowchart of study procedure.

Participants completed the online alcohol consumption diary before returning to the laboratory for the second study session. Reminders and links to access the diary were automatically sent to participants every day. All participants returned for the second session 1 week after the first session. After arriving, participants were instructed to complete the free and cued recall tasks following the same procedure as in the first study session. Thereafter, participants were fully debriefed, paid, and thanked for their time (see Figure 1 for a schematic outline of the study procedure).

### 2.5. Coding

All free recall accounts were transcribed for coding purposes. Free recall completeness and accuracy were measured in this study. Completeness was measured by counting the total number of details recalled by the participant. Accuracy was calculated by dividing the number of accurate details recalled by the total number of details recalled. Details were coded using the same free recall procedure as Jaffe (2018; see Table 1). Every individual mention or description of a person or object was coded upon their first mention and repetitions were not coded again. A detail was deemed accurate if it corresponded with the film (e.g., three women driving in a car), or inaccurate if the detail did not correspond with the film (e.g., four women driving in a car, three women driving in a van, etc). Twelve of the 60 (20%) free recall transcripts from participants taking part in both sessions were coded by two additional independent and naïve raters to ensure that coding was carried out consistently. Interclass correlation analysis revealed a high level of consistency between raters (*ICC >* 0.750, *p* < 0.001, for frequency of accurate and inaccurate details).

**Table 1:** Free recall coding instructions (originally from Jaffe, 2018).

Step	Instruction
1	Code the number of objects/people mentioned. Any mention of an object (regardless of the term used) should be included. For example, if a participant stated there was a “veil” in the room, you would indicate, YES, the “curtain” was mentioned.
2	For each object that is mentioned, code the number of accurate details. For each person that is mentioned, code the number of accurate details and actions. Only count each action once (assign it to one subject). Only consider observable details (not judgments or inferences). Determine accuracy by referring to the relevant photos and the film clip
3	For people in the film, locations/possessions should only be considered a detail when not referring to a separate film-object.
4	Determine whether there was any inaccurate information reported. Count up and indicate the total number of inaccurate units.
5	If you have other questions or are unsure how to code something, make a note to Anna in the text box at the end of the survey. (If you say you were unsure whether to code something, be sure to indicate whether you counted it or not in the response you submitted.)
6	Do not code emotional reactions, judgments, personal anecdotes, or other editorializing. Do not code anything unrelated to the actual content of the film (e.g., tasks completed as part of the study, grammatical and spelling errors, how the free recall response was organized etc).

## 3. Results

### 3.1. Free recall performance

We performed a mixed-factor ANOVA with a repeated measures factor of Session (1 vs. 2), a between-groups factor of Condition (Alcohol-Exposed vs. Placebo-Control), and their interaction. Descriptive statistics are presented in Table 2 across outcome measures. A summary of main effects and interaction is presented in Table 3.

**Table 2:** Means and (standard deviations) across conditions – free recall.

		Total Details		Accurate Details		Inaccurate Details		Percentage Accurate	
		*M*	SD	*M*	SD	*M*	SD	*M*	SD
Session 1	Alcohol	25.10	7.24	23.23	6.98	1.87	1.22	92.32	5.37
	Placebo	27.10	6.22	24.70	5.92	2.40	1.79	91.17	6.61
Session 2	Alcohol	28.90	7.38	26.67	7.00	2.23	1.45	92.22	4.55
	Placebo	27.93	6.74	25.33	6.34	2.60	2.04	90.71	7.54

**Table 3:** Summary of main effects and interactions.

	Total Details			Accurate Details			Inaccurate Details			Percentage Accurate		
	*F*	*p*	*η_p_* ^2^	*F*	*p*	*η_p_* ^2^	*F*	*p*	*η_p_* ^2^	*F*	*p*	*η_p_* ^2^
Session	**15.47**	**<0.001**	**0.211**	**11.26**	**0.001**	**0.163**	1.82	0.183	0.030	0.11	0.741	0.002
Condition	0.94	0.760	0.002	0.02	0.967	0.000	1.45	0.233	0.024	0.97	0.328	0.016
Interaction	**6.34**	**0.015**	**0.099**	**5.34**	**0.024**	**0.084**	0.16	0.693	0.003	0.05	0.830	0.001

Significant main effects and interactions are in bold. All degrees of freedom = 1,58.

Significant main effects of Session were found on Total Details recalled (Session 1 = 26.10; Session 2 = 28.42) and Accurate Details recalled (Session 1 = 23.97; Session 2 = 26.00). There were no significant effects of Session on Inaccurate Details recalled nor Percentage of Accurate Details recalled.

There were no significant main effects of Condition on any of the outcome variables (all *F*s ≤ 1.45, all *p*s ≥ 0.233).

**Figure 2:**
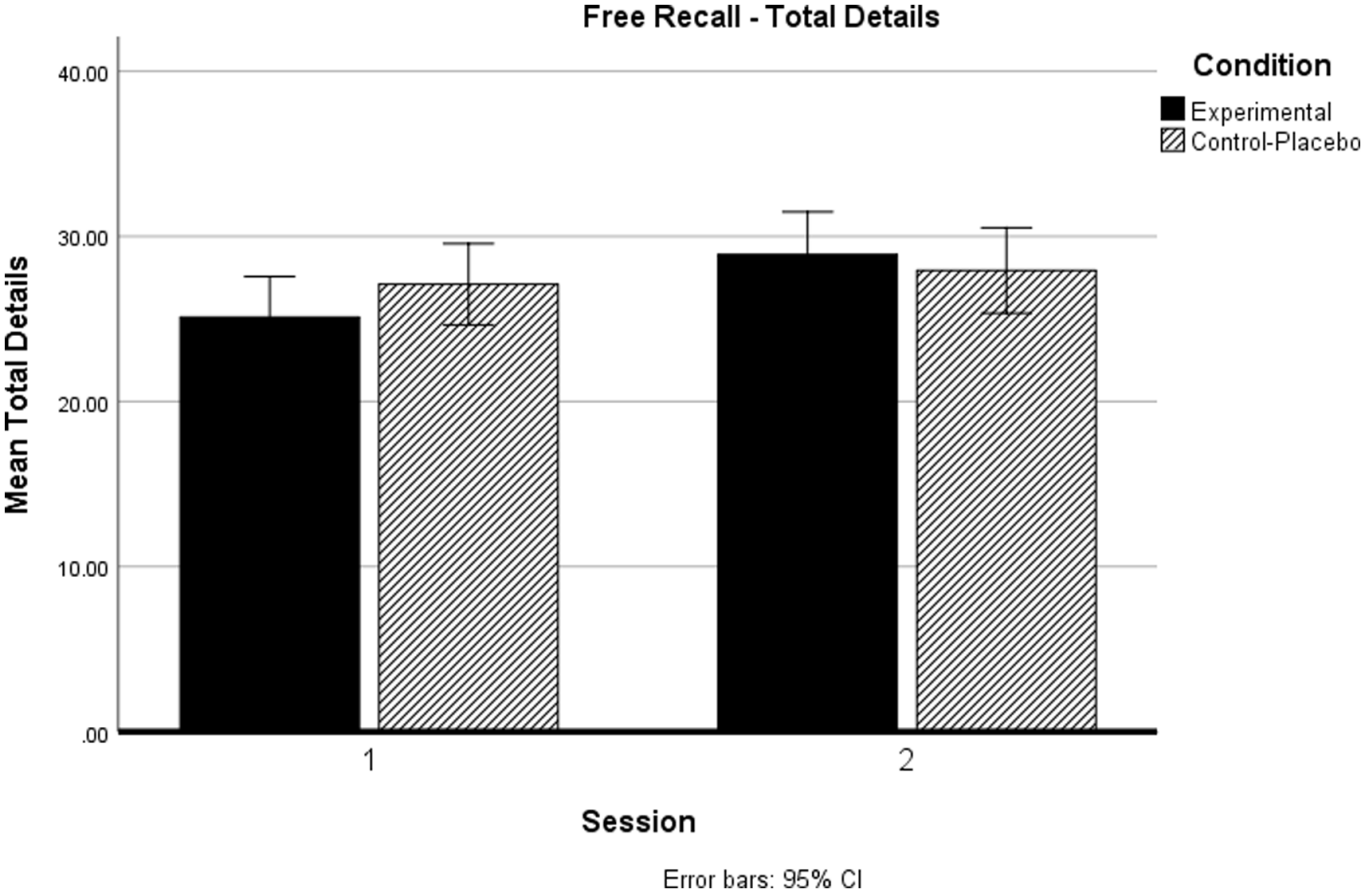
Session × Condition interactions on Total Details recalled.

**Figure 3:**
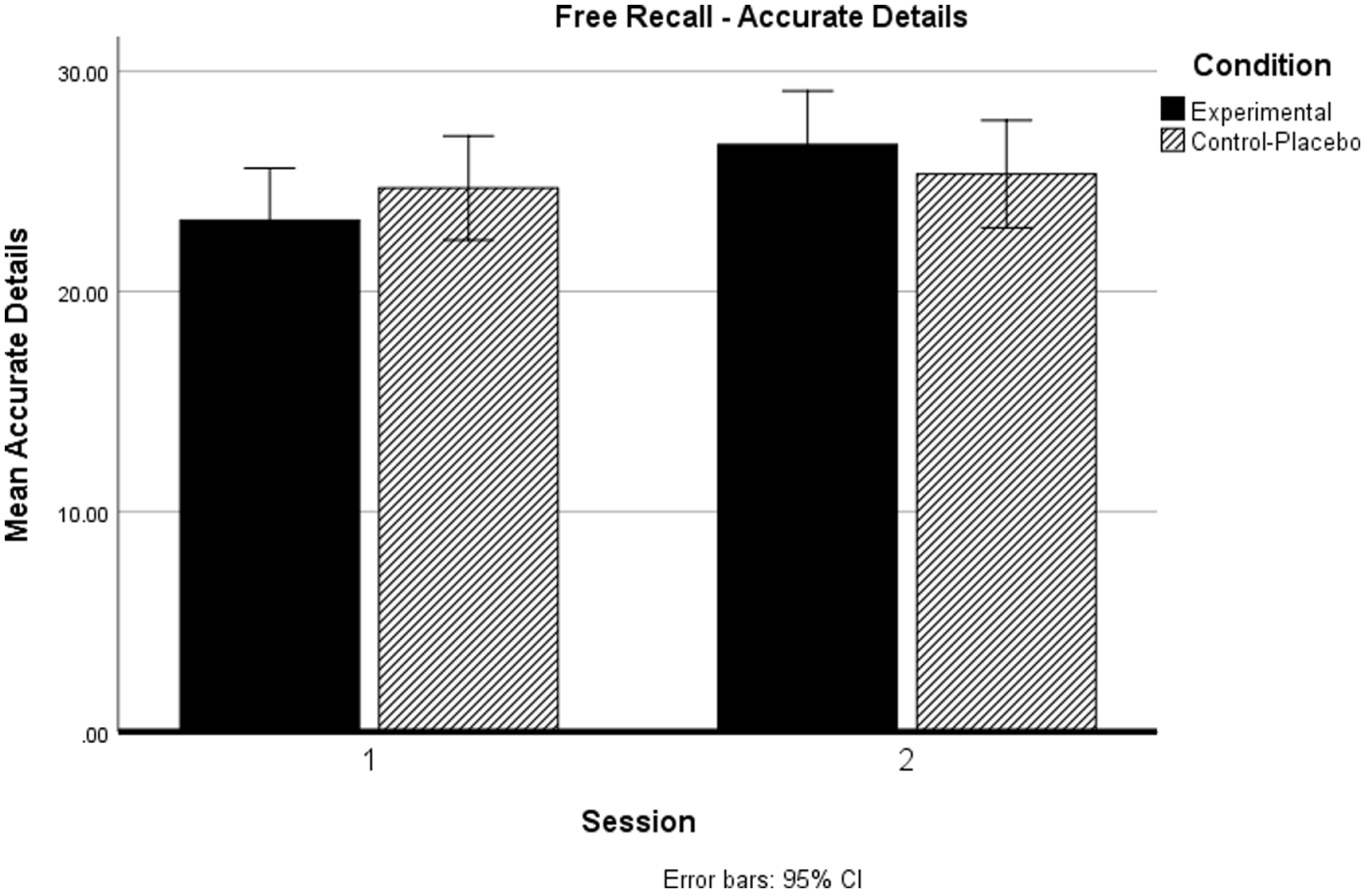
Session × Condition interactions on Accurate Details recalled.

Crucially, we observed significant Session × Condition interactions on Total Details recalled and Accurate Details recalled. These interactions are illustrated in Figure 2 (Total Details) and Figure 3 (Accurate Details).

Considering Total Details recalled, simple main effects analysis revealed that there were no significant differences in Total Details recalled by-group at Session 1 (*p* = 0.256), nor at Session 2 (*p* = 0.598). Importantly, our Placebo-Control group showed no change in Total Details recalled between Session 1 and Session 2 (*p* = 0.321), whereas our Alcohol-Exposed experimental group showed improved performance from (impaired) Session 1 to (unimpaired) Session 2 (*p* < 0.001).

An almost identical pattern was observed for Accurate Details recalled. Simple main effects analysis revealed that there were no significant differences in recall by group at Session 1 (*p* = 0.384), nor at Session 2 (*p* = 0.443). Importantly, our Placebo-Control group showed no change in Accurate Details recalled between Session 1 and Session 2 (*p* = 0.463), whereas our Alcohol-Exposed experimental group showed improved performance from (impaired) Session 1 to (unimpaired) Session 2 (*p* < 0.001).

### 3.2. Cued recall

We considered the relative frequency of “answer types” (“remember,” “know,” “do not know”) regardless of response accuracy of “remember” and “know” judgments. This data was analyzed *via* Pearson’s chi-square. Table 4 displays a contingency based on experimental Session and Condition vs. Answer Types.

**Table 4:** Contingency of cued recall answer types across conditions.

		“Remember”	“Know”	“Do not Know”
Session 1	Alcohol	219	148	113
	Placebo	214	218	96
Session 2	Alcohol	199	165	116
	Placebo	187	188	93

Analysis suggested that there was no relationship between Session and answer distribution [*χ*^2^ (2) = 1.09, *p* = 0.579; Cramer’s *V* = 0.024]. However, there was an association between Condition and answer distribution [*χ*^2^ (2) = 15.55, *p* < 0.001; Cramer’s *V* = 0.089]. The Alcohol-Exposed group reported fewer “remember” and fewer “know” responses than would have been expected at chance, and more “do not know” responses than would have been expected at chance. Contrastingly, the Placebo-Control group demonstrated far more “know” responses than expected at chance, and fewer “do not know” responses than expected at chance.

As with free recall, we considered the number of responses given by participants based on the independent and combined effects of Session and Condition *via* mixed-factor ANOVA. Descriptive statistics are presented in Table 5, and main effects and interactions are summarized in Table 6.

**Table 5:** Means and (standard deviations) across conditions – cued recall.

		“Remember”	Correct “Remember”	“Know”	Correct “Know”	“Do not Know”
Session 1	Alcohol	7.40 (2.51)	4.93 (2.05)	4.87 (2.36)	1.30 (1.26)	3.73 (2.38)
	Placebo	6.66 (2.55)	4.31 (1.75)	6.59 (3.06)	2.24 (1.48)	2.77 (1.74)
Session 2	Alcohol	6.53 (2.78)	4.40 (2.01)	5.50 (2.73)	2.23 (1.55)	3.87 (2.36)
	Placebo	6.38 (2.69)	4.41 (1.97)	6.45 (3.72)	2.49 (2.08)	3.07 (2.42)

**Table 6:** Main effects and interactions – cued recall.

	Session			Condition			Interaction		
	*F*	*p*	*η_p_* ^2^	*F*	*p*	*η_p_* ^2^	*F*	*p*	*η_p_* ^2^
“Remember”	3.74	0.058	0.062	0.53	0.471	0.009	1.00	0.321	0.017
Correct “Remember”	1.65	0.204	0.028	0.40	0.528	0.007	3.62	0.062	0.060
“Know”	0.76	0.388	0.013	3.36	0.072	0.056	1.84	0.181	0.031
Correct “Know”	**7.77**	**0.007**	**0.120**	2.47	0.122	0.041	3.15	0.081	0.052
“Do not Know”	1.15	0.288	0.019	2.64	0.109	0.044	0.17	0.681	0.003

Significant main effects and interactions are in bold.

The only result of note was that the number of correct “know” responses increased from Session 1 to Session 2, driven by an increase in this proportion of responses by the Alcohol-Exposed group (as suggested by the marginal Session × Condition interaction).

### 3.3. Confidence data

Participants were also asked to provide ordinal “confidence ratings” for each of their answers. We performed a series cumulative linked mixed effects model analyzes on these data (see, for example, Hand et al., 2022; Taylor et al., 2022), to explore the fixed and combined effects of Session, Condition, and Answer Accuracy. Descriptive statistics are presented in Table 7.

**Table 7:** Mean confidence ratings across session, condition, and answer accuracy.

		Correct		Incorrect	
		*M*	SD	*M*	SD
Session 1	Alcohol	81.63	22.21	31.57	32.01
	Placebo	72.29	27.31	37.48	32.17
Session 2	Alcohol	74.84	25.25	29.77	31.45
	Placebo	73.37	26.84	33.31	30.98

The main effect of Session on confidence was significant [*χ*^2^ (1) = 6.04, *p* = 0.014]; confidence was higher in Session 1 (55.74) than in Session 2 (52.82). The main effect of Condition on confidence was non-significant [*χ*^2^ (1) < 1]. The main effect of Answer Accuracy on confidence was significant [*χ*^2^ (1) = 33.48, *p* < 0.001]; confidence was higher alongside Correct Answers (75.53) than Incorrect Answers (33.03).

There was a significant interaction between Condition and Accuracy [*χ*^2^ (1) = 12.01, *p* < 0.001]. Follow-up comparisons revealed that, when giving *wrong* answers, the Placebo-Control group were more confident (35.40) than the Alcohol-Exposed group (30.67; *p* = 0.006). However, when giving *correct* answers, the Alcohol-Exposed group were more confident (78.23) than the Placebo-Control group (72.83; *p* = 0.010).

There was no significant interaction between Session and Answer Accuracy [*χ*^2^ (1) = 1.26, *p* = 0.261].

There was a significant interaction between Session and Condition [*χ*^2^ (1) = 8.41, *p* = 0.004]. Follow-up comparisons revealed that for the Placebo-Control group there was no difference in confidence ratings between Session 1 (54.88) and Session 2 (53.34; *p* = 0.415). However, for the Alcohol-Exposed group, confidence was significantly higher at (exposed) Session 1 (56.60) than (non-exposed) Session 2 (52.30; *p* = 0.026).

The three-way interaction between Session, Condition, and Answer Accuracy was non-significant [*χ*^2^ (1) = 2.72, *p* = 0.099].

### 3.4. Take-home messages

1. Under free recall conditions, providing individuals who immediately drank *after* they encountered a negative event with a delayed recall opportunity when sober again led to additional details and more complete recall overall. There was no significant difference in accuracy rates between Sessions 1 and 2 for the Alcohol-Exposed group, suggesting that the increase in recall quantity did not coincide with a decrease in quality. This is in line with previous literature showing that a subsequent retrieval attempt can be beneficial (e.g., LaRooy et al., 2013; Odinot et al., 2013).
2. Alcohol after encoding led to fewer “remember” and “know” responses and more “do not know” responses compared to chance. Thus, post-encoding alcohol consumption seems to make one less certain about one’s memory recollection. We did not observe the anticipated decrease in “remember” responses and increase in “know” responses in the Alcohol-Exposed group. The Placebo-Control group seemed more confident in their memory as indicated by higher-than-chance “know” responses and fewer than-chance “do not know” responses. Thus, alcohol intake after having witnessed a negative event appears to makes one more uncertain about one’s memory recollection.
3. In line with the idea that a second retrieval attempt is beneficial in terms of the completeness of the information obtained, participants who were intoxicated during the first retrieval attempt made more correct “know” responses during the second delayed retrieval attempt when sober again.
4. Alcohol-Exposed individuals did become less confident as time passed. Confidence faded from session 1 to session 2. Unexpectedly, when giving wrong answers, the Placebo-Control group was more confident than the Alcohol-Exposed group; when giving correct answers, the Alcohol-Exposed group was more confident than the Placebo-Control group.

## 4. Discussion

This is the first experimental study examining the effects of post-encoding alcohol intoxication on immediate and delayed episodic memory recall for a traumatic event in a sample of heavy drinkers. We found no significant differences in episodic memory accuracy or completeness between the Alcohol-Exposed and Placebo-Control groups in sessions 1 or 2. However, the alcohol group demonstrated an improvement in episodic memory completeness between sessions. Individuals who drank alcohol immediately after witnessing the traumatic car accident recalled more details after a week’s delay compared to directly after the event. Placebo-Control group memory performance did not change over time. The placebo group demonstrated more familiarity-based processing (i.e., more “know” responses than at chance) than the alcohol group. Again, the placebo group did not show a significant change in performance between sessions, whereas the alcohol group displayed an increase in familiarity-based processing (i.e., more correct “know” responses) over time. The increase suggests that post-traumatic alcohol intoxication may have a specific effect on familiarity-based processing, affecting automatic sensory memory rather than the conscious recollection of contextual details.

### 4.1. Episodic memory recall

In terms of episodic memory accuracy and completeness, the present findings are broadly consistent with previous research on pre-encoding alcohol intoxication on eyewitness event recall. While some studies found that pre-encoding intoxication impairs episodic memory accuracy (Altman et al., 2018), most showed that accuracy is unaffected (Colloff and Flowe, 2016; Flowe et al., 2017; Gawrylowicz et al., 2019). Unlike accuracy, episodic memory completeness is affected by pre-encoding alcohol exposure. Multiple studies suggest that alcohol prior to encoding leads to less-detailed accounts of the to-be-remembered event (e.g., Schreiber Compo et al., 2011; Hagsand et al., 2013; Flowe et al., 2016); our findings on the effects of post-encoding alcohol intoxication on event recall are somewhat consistent in this respect. Although, we did not find group differences between the Alcohol-Exposed and Placebo-Control groups during sessions 1 or 2, we did find that Alcohol-Exposed participants recalled more and more accurate details during the delayed repeated retrieval opportunity.

This finding is important as it supports the practical implication that witnesses and victims who drank *after* experiencing trauma may benefit from a repeated delayed interview. That repeated interviewing can lead to the recall of reminiscent details in sober as well as intoxicated individuals has been empirically established, for example, LaRooy et al. (2013) found that mock-witnesses who had been intoxicated during encoding and sober during the repeated retrieval attempt, recalled 20% of new details. This study extends those findings to individuals who drank alcohol *after* they witnessed a traumatic event. Our findings suggest that they might benefit even more from repeated interviewing than those who did not drink alcohol after trauma. Given that a disproportionate number of offenses happen in places where alcohol is sold and consumed (i.e., pubs and clubs) (Leonard et al., 2002), it is not unlikely that witnesses and victims might consume alcohol shortly after they have witnessed a crime. Police should be aware that a subsequent retrieval attempt might be particularly valuable for those individuals.

Experimental studies that have examined post-encoding alcohol intoxication on episodic memory recall are rare. Gawrylowicz et al. demonstrated that post-encoding intoxication reduced the likelihood of accepting misleading post-event information compared to a placebo group. While this does not test episodic memory completeness, it suggests that post-encoding intoxication should be considered independently from pre-and peri-traumatic intoxication. Schreiber-Compo et al. found that post-encoding intoxication had no effect on episodic memory accuracy or completeness. However, their manipulation of alcohol intoxication differed from the present study; participants watched a film while intoxicated or sober and then recalled the content in the same state and 1 week later in a sober or intoxicated state. Intoxicated participants recalled most details when the recall happened immediately and did not benefit from state-dependent recall. In the present study, participants were only intoxicated *after* watching a film, and not again 1 week later. Methodological differences such as these may explain the difference in findings. Going forward, future research examining alcohol-related effects on episodic memory should consider the paradoxical effects alcohol may have on encoding, consolidation, and recall, and try to disentangle its attenuating and facilitating effects on event recall.

Our study found that post-encoding alcohol intoxication did not facilitate immediate event recall, but individuals showed improved recall over time, after a 1 week delay. Retrograde facilitation may explain this paradoxical finding. It could be argued that alcohol intoxication interrupts neural processes in the hippocampus critical for forming new memories (Bliss and Collingridge, 1993; Söderlund et al., 2007). Post-encoding intoxication immediately following an event would therefore enhance one’s ability to “block-out” post-event intrusions and any other interfering new information and allow one to process memory for the event more fully, thereby increasing subsequent memory recall completeness.

### 4.2. Experiential recollection (remembering/knowing)

In comparison to episodic memory accuracy and completeness, there is a smaller selection of studies that investigated the effects of alcohol intoxication upon experiential recollection. Curran and Hildebrandt (1999) and Bisby et al. (2010) found that alcohol intoxication impaired “remember” responses, whereas “know” responses remained unaffected. Although our methodology varies from theirs, in that Curran et al. and Bisby et al. only tested the effects of pre-encoding rather than post-encoding intoxication, our findings do not support the notion that alcohol impairs in particular conscious recollection of contextual details more so than that of unconscious familiarity-based processes.

The present study demonstrates that post-encoding alcohol intoxication results in improved familiarity-based processing (more correct “know” responses) over the course of 1 week. A tendency toward familiarity-based processing alone, rather than recollection-based processing, implies that post-encoding alcohol intoxication might have specific effects on episodic memory related to automatic unconscious sensory recollection. While conscious contextual representations are critical for the development of episodic memory, sensory representations are also important and contribute to overall episodic memory quality (Brewin, 2014). Considering the results of the free and cued recall tests together, post-encoding intoxication may be targeting sensory rather than contextual representations, resulting in significant but modest influences on overall episodic memory.

### 4.3. Critical reflection

The present study offers several novel features, such as manipulating post-encoding rather than pre-encoding intoxication and recruiting a community-based sample of individuals who drink heavily. However, the present study has limiting factors, which have been measured and mitigated where possible. For example, while breathalyzer data was used to measure acute alcohol intoxication in the first session, self-report measures of intoxication were not collected. Higher self-reported intoxication can impact confidence in test performance, regardless of actual intoxication (Assefi and Garry, 2003). While this is certainly a limitation, data from the breathalyzer measurements confirm sufficient intoxication in the Alcohol-Exposed condition. Measures such as disguising the taste of beverages and providing an engaging distractor task helped to improve the efficacy of the placebo, with nearly all participants providing anecdotal accounts of feeling intoxicated during the experiment. Furthermore, previous research suggests that having a history of alcohol blackouts affects episodic memory performance, causing greater impairment in memory tasks when also intoxicated (Wetherill and Fromme, 2016). While history of blackouts was recorded, the present study was unable to account for blackouts when analyzing the effect of post-traumatic alcohol intoxication upon episodic memory performance. This is due to the decision to recruit from a sample of heavy-drinking individuals from the local community, whereby most participants reported some history of alcohol blackouts. While not intentional, this further supports the recruitment rationale compared to convenience sample of university students, highlighting influential variables that differ to the general population.

A further limitation is that we did not ensure that the event was equally stressful for both groups. This is particularly important given recent research findings demonstrating that acute stress does not result in general memory impairment or improvement (see for a review Schwabe et al., 2022), but that the relationship is complex and likely time dependent. That is depending on *when* the stress is experienced different memory systems and their interchange are impacted, leading to recall improvements as well as deficits, depending on the exact timing of when the stressor is experienced/administered, the to-be-learned material is encoded, and then subsequently retrieved. For example, Krenz et al. (2021) showed that noradrenergic arousal shortly after encoding changed the systems consolidation dynamic in their participants. In contrast to the placebo group, participants who received a α2-adrenoceptor antagonist yohimbine (YOH) shortly before encoding showed increased hippocampal activity and decreased neocortical activity over a time frame of 28 days. Compared to the placebo group, the YOH group showed less memory decline over time. Future research would benefit from trying to disentangle how stress and alcohol together impact episodic memories, as these two factors often coincide (e.g., eyewitness testimony). Furthermore, it would be helpful to study whether similar to stress alcohol’s effects on episodic memory are lasting (i.e., lasting longer than a week).

A caveat of the current design is that all participants engaged in an immediate recall test, and it is well documented in the empirical literature that an early retrieval attempt can lead to enhanced memory at subsequent retrieval attempts (e.g., Krix et al., 2014; Ma et al., 2022). It could be argued that this initial recall test facilitated the activation of the encoded material and subsequent consolidation, thereby masking any alcohol-related effects. An ideal design would manipulate retrieval retention interval so that the time-dependent effects of alcohol on episodic memory performance can be more systematically studied.

Pertaining to testing “remember/know” responses, it is important to acknowledge that the participant’s understanding of these terminologies might differ from the researcher’s (see Umanath and Coane, 2020 for a critical reflection). To avoid problems with clarity and confusion on the part of participants in future studies, research designs should implement additional training, such as providing a practice phase and/or additional examples on how to use “remember/know” responses. Post-tests can also be applied to find out whether participants used “remember/know” responses as intended by the researcher (Umanath and Coane, 2020).

Our findings contribute to the understanding of how post-traumatic alcohol intoxication may affect the development of PTSD, particularly among heavy-drinking individuals. Reliance on familiarity-based processing following post-traumatic intoxication may influence overall episodic memory quality. Reliance on familiarity-based processing for traumatic memories also affects mental health, as recognizing details from a traumatic event without context can trigger a fear response similar to re-experiencing the traumatic event (Brewin, 2007). Over time, this might contribute to mental health issues such as hypervigilance and avoidance (Halligan et al., 2003), potentially developing into PTSD. The effects of psychological trauma on episodic memory are therefore critical to mental health, with efforts to reconstruct episodic memory being incorporated into treatments for PTSD (Ehlers et al., 2005). Whereas previous studies have demonstrated a relationship between pre-traumatic alcohol intoxication and the development of PTSD symptoms (Bisby et al., 2009), the current study is the first to explore how post-traumatic alcohol intoxication may influence episodic memory (and thereby potentially affect the development of PTSD). Our findings are particularly relevant to heavy-drinking individuals, who are more likely to engage in post-traumatic alcohol use, experience more intense trauma symptoms, and are more likely to be diagnosed with PTSD (Kaysen et al., 2007; Olff et al., 2007). It is recommended that future work and therapies focus on the propensity of heavy-drinking individuals to engage in familiarity-based processing following post-traumatic alcohol intoxication, to help mitigate the development of PTSD symptoms.

### 4.4. Conclusion

Alcohol has complex and paradoxical effects on episodic memory performance. Depending on the dosage, the emotional content of the to-be-remembered event, the timing of alcohol consumption, and the timing of the recall attempt, event recall might be impaired or facilitated. We found that post-encoding alcohol intoxication led to more complete memory accounts about a witnessed traumatic car accident when elicited after a 1 week delay compared to immediately. The relative improvement in episodic memory completeness in the alcohol condition is consistent with previous research on pre-encoding alcohol-related memory effects. We also found that post-traumatic alcohol intoxication improved familiarity-based processing; that is, elements of episodic memory related to sensory details and not specifically tied to contextual details. While these findings are important in themselves, they are highly relevant when it comes to informing and treating individuals who consume alcohol to cope with negative life experiences. Particularly, those who use alcohol to cope with negative memories resulting from trauma. Our findings suggest that immediate alcohol consumption after an observed traumatic event does not help to forget – rather, it leads to more complete recollection over time. Immediate alcohol intake after trauma may also lead to the later recollection of more sensory details, which potentially could lead to a manifestation of PTSD like symptoms. Concerning investigative interviewing of witnesses and victims, the present finding suggests that police should provide individuals with a delayed repeated retrieval opportunity, especially in cases where the witness and/or victim drank alcohol after the incident has happened, as this might lead to more detailed accounts.

## Data availability statement

The datasets presented in this study can be found in online repositories. The names of the repository/repositories and accession number(s) can be found at: Open Science Framework https://osf.io/pwqd5/?view_only=2a0bca0548924353b907896cbf796d11.

## Ethics statement

The studies involving human participants were reviewed and approved by Glasgow Caledonian University. The patients/participants provided their written informed consent to participate in this study.

## Author contributions

All authors listed have made a substantial, direct, and intellectual contribution to the work and approved it for publication.

## Funding

The research was funded by a University PhD Research Studentship, from Glasgow Caledonian University. Project Title: An Exploration of the influence of alcohol on memory for traumatic events. Project reference number: S2017SHLS009. Awarded to JG and KL.

## Conflict of interest

The authors declare that the research was conducted in the absence of any commercial or financial relationships that could be construed as a potential conflict of interest.

## Publisher’s note

All claims expressed in this article are solely those of the authors and do not necessarily represent those of their affiliated organizations, or those of the publisher, the editors and the reviewers. Any product that may be evaluated in this article, or claim that may be made by its manufacturer, is not guaranteed or endorsed by the publisher.
